# Failure to demonstrate effects of interruptions on diagnostic reasoning: three experiments

**DOI:** 10.1186/s12909-022-03212-1

**Published:** 2022-03-16

**Authors:** Mai Alajaji, Nada Saleh, Ali Hassan AlKhulaif, Silvia Mamede, Jerome I. Rotgans, Hatouf Sukkarieh, Nouf AlHarbi, Mohi Eldin Magzoub, Henk G. Schmidt

**Affiliations:** 1 College of Pharmacy, King Saud bin Abdulaziz University for Health Sciences, King Abdullah International Medical Research Center, Riyadh, Saudi Arabia; 2 College of Medicine, King Saud bin Abdulaziz University for Health Sciences, Ar Rimayah, Riyadh, 14611 Saudi Arabia; 3 Emergency Medicine Department, King Abdullah bin Abdulaziz University Hospital, Riyadh, 11564 Saudi Arabia; 4 Erasmus University, Institute of Medical Education Research Rotterdam, Erasmus Medical Centre, and Department of Psychology, Erasmus University Rotterdam, Rotterdam, P.O. Box 1738, 3000 DR The Netherlands; 5 Medical Education Research and Scholarship Unit, Lee Kong Chian School of Medicine, Nanyang Technological University, Singapore City, 308232 Singapore; 6 College of Medicine, Alfaisal University, Takhassusi St, Riyadh, 11533 Saudi Arabia; 7 College of Medicine and Health Sciences, United Arab Emirates University, P.O. Box 17666, Al Ain, United Arab Emirates; 8 Department of Psychology, Erasmus University Rotterdam, P.O. Box 1738, 3000 DR Rotterdam, The Netherlands

**Keywords:** Diagnostic error, Cognition, Internal medicine, Emergency medicine

## Abstract

**Background:**

Diagnostic error is a major source of patient suffering. Researchshows that physicians experience frequent interruptions while being engaged with patients and indicate that diagnostic accuracy may be impaired as a result. Since most studies in the field are observational, there is as yet no evidence suggesting a direct causal link between being interrupted and diagnostic error. Theexperiments reported in this article were intended to assess this hypothesis.

**Methods:**

Three experiments were conducted to test the hypothesis that interruptions hurt diagnostic reasoning and increase time on task. In the first experiment (*N* = 42), internal medicine residents, while diagnosing vignettes of actual clinical cases were interrupted halfway with a task unrelated to medicine, solving word-spotting puzzles and anagrams. In the second experiment (*N* = 78), the interruptions were medically relevant ones. In the third experiment (*N* = 30), we put additional time pressure on the participants. In all these experiments, a control group diagnosed the cases without interruption. Dependent variables were diagnostic accuracy and amount of time spent on the vignettes.

**Results:**

In none of the experiments interruptions were demonstrated to influence diagnostic accuracy. In Experiment 1: Mean of interrupted group was 0.88 (SD = 0.37) versus non- interrupted group 0.91 (SD = 0.32). In Experiment 2: Mean of interrupted group was 0.95 (SD = 0.32) versus non-interrupted group 0.94 (SD = 0.38). In Experiment 3: Mean of interrupted group was 0.42 (SD = 0.12) versus non-interrupted group 0.37 (SD = 0.08). Although interrupted residents in all experiments needed more time to complete the diagnostic task, only in Experiment 2, this effect was statistically significant.

**Conclusions:**

These three experiments, taken together, failed to demonstrate negative effects of interruptions on diagnostic reasoning. Perhaps physicians who are interrupted may still have sufficient cognitive resources available to recover from it most of the time.

## Background

Medical errors are among the major causes of mortality and morbidity worldwide, with an estimated annual medical error-associated death rate of 163,156 in the United States [1] and 25,000 in the United Kingdom [2]. Diagnostic error can be defined as “a diagnosis that was unintentionally delayed, wrong, or missed, as judged from the eventual appreciation of more definitive information” [3]. Studies conducted in the 80’s and 90’s estimated the rate of diagnostic error in clinical medicine to be approximately between 15 and 39% [4, 5], which was corroborated by autopsy-based studies [6, 7], though only capturing a fraction of the consequence of diagnostic errors as most will not result in death and diagnostic errors remain under-reported. This rate is greatest in internal medicine where the spectrum of clinical problems, and subsequent diagnostic decisions, is significantly greater than other specialties [8].

Many studies reporting on diagnostic errors are retrospective and/or autopsy-based. An example of such a study in internal medicine was conducted by Graber and Franklin [3]*.* They proposed that diagnostic error can been etiologically categorized into no-fault errors, system-related errors and cognitive errors with each category contributing 7, 19 and 28% respectively. On the other hand, Croskerry [9] attributes a considerable and important subset of diagnostic errors to cognitive dispositions to respond that are associated with failures in perception, heuristics and/or biases. Several studies have been done looking into the sources of cognitive diagnostic errors. Recently, time pressure has been shown to negatively affect diagnostic accuracy [10]. This effect was attributed to increased stress and a reduction in the number of diagnostic hypotheses generated under time pressure [11]. The latter corroborates Graber and colleagues’ autopsy-based study in which they report that cognitive errors, especially those resulting from faulty hypotheses synthesis and deficiencies in approaching the problem, are a common cause of diagnostic error [3].

### Effects of interruptions on diagnostic error and time on task

The studies presented in this article focus on another potential source of diagnostic error: Being interrupted by extraneous events while diagnosing a patient. Physicians experience frequent interruptions of various types in a broad range of settings during tasks related to patient care [12]. Interruptions include human communication and electronic ones [13, 14]. Emergency physicians and primary-care physicians are interrupted on average 9.7 and 3.9 times per hour, respectively [15]. A recent review estimates that the average number of interruptions ranged from 0.3 to 13.9 times per hour and up to 20 times per hour in the emergency department [16]. One would intuitively assume that these interruptions may affect physicians’ reasoning, causing mistakes. They hinder healthcare effectiveness and can negatively affect performance. This includes an increase in the incidence of medical errors as concluded by the Agency for Healthcare Research and Quality [17]. Indeed, research in psychology suggests that interruptions disrupt human cognition [5, 18]. It is not known, however, whether interruptions in fact cause diagnostic errors.

Research on interruptions in clinical practice has grown in recent years, triggered by evidence of the deleterious consequences of interruptions in other domains, such as aviation [19]. Reducing interruptions has been proposed as an important intervention to minimize medical errors. Nevertheless, it is recognized that evidence of an association between interruptions and errors in areas other than medication dispensing is insufficient [17, 20]. Interruptive irrelevant communications, for example, have been found to occur in rates of 11.15 per hour in an emergency department [21] and of 3.48 per surgical procedure [22]. Most interruptions have the form of a “break-in-task”, i.e., an event that not only diverts the attention of the physician, but results in switching to a new task with the assumption that the initial task will be resumed [15].

Despite the evidence showing the magnitude of interruptions physicians experience, it is not clear whether these interruptions lead to diagnostic errors. Studies so far have had a descriptive or observational nature and have focused on the adverse effects of interruptions on performance of surgical [22, 23] or therapeutic [24, 25] procedures. Nevertheless, experimental studies may yield insights into our understanding of the effects of interruptions on diagnostic accuracy, as well as clinical reasoning. Recently, Monteiro et al. [26] showed that interruptions have a negative effect on response time but not on accuracy. Psychology research has shown, however, that interruptions disrupt cognitive processes and indeed can induce errors. The limited capacity of our mental resources restricts the possibility of accurately performing two tasks simultaneously at least when they are performed non-automatically [18, 27]. Interruption and switching to another task may affect performance by interfering with memory of information encountered in the original task, information that is essential to resume the original task effectively [28]. Interruptions result in a break preventing the continuation of the task at hand and forcing the individual to switch attention to another mental, and potentially physical, task. Resuming the original task and retrieving the information pertinent to it can be negatively affected by interruptions by decreasing situational awareness [19] and time needed to resume primary task [29]. Limited processing capacity and forgetting, shown to affect reasoning in other domains, are likely to also disrupt physicians, but whether and how this occurs is unknown.

### Purpose of the present studies

With the exception of the Monteiro and colleagues' [26] study, effects of interruptions in the domain of medicine have only been studied using descriptive or observational approaches. Therefore, causal evidence of the disruptive effect of interruptions on diagnostic decision-making is presently limited. In this article, we report three experimental attempts to study the effects of interruptions on diagnostic reasoning in residents. In the first experiment, emergency and internal medicine residents, while diagnosing vignettes of actual clinical cases were interrupted halfway with a task unrelated to medicine, solving anagrams. In the second experiment, the interruption was more lifelike. And in the third experiment, we put time pressure on the participants. In each of the experiments, and following the suggestions of the psychology literature, we hypothesized that under interruption conditions, diagnostic accuracy would decrease whereas time-on-task would increase.

## Experiment 1

In a randomized trial, emergency and internal medicine residents were confronted with eight vignettes of actual clinical cases, presented on a computer screen in a counterbalanced order. They were required to provide a most likely diagnosis for each of the cases. Time on task was recorded. Half of the participants were interrupted by a second task, a puzzle or an anagram, while diagnosing the cases. Although they were encouraged to work fast, no time restrictions were applied.

### Methods

#### Participants

A convenience sampling technique was used. Forty residents were invited to participate. Of these, 37 eventually showed up. Therefore, five additional subjects were recruited at a later stage. Participants were 15 female and 27 male emergency and internal medicine residents from an academic hospital in Saudi Arabia. Department heads were contacted to recruit residents from the third and fourth year of their program. Median age was 28 years (SD = 1.87). Mean number of years of clinical practice was 3.62 (SD = 1.17). No significant differences in gender, age and clinical practice were observed between treatment and control group. Ethical approval to conduct the study was granted by the institutional review board of the King Abdullah International Medical Research Center (KAIMRC), Riyadh, Saudi Arabia.

Participants were informed briefly about the experiment by the principal investigator, and written consent was obtained. To avoid influencing the data, the study purpose was not disclosed to the participants. The participants were randomly allocated to the conditions of the experiment.

#### Materials

The study included *eight written clinical vignettes* selected from a set of cases used by Mamede et al. [30] that were of similar difficulty. The cases were selected based on the mean diagnostic scores obtained by the residents in those studies and concerned the following diseases: Hyperthyroidism; pseudomembranous colitis; Addison’s disease; inflammatory bowel disease; acute viral hepatitis; liver cirrhosis; acute appendicitis, and aortic dissection. Each case was presented in English and consisted of a brief description of a patient’s medical history, signs, symptoms, the outcome of physical examination and tests results. Table 1 presents an example of a case used in the study. All cases were based on real patients with a confirmed diagnosis. Two Saudi consultants reviewed the cases ensuring their appropriateness for the local context.

**Table 1:** Example of a case used in all three experiments

*A 32-year-old woman consulted the doctor with complaints of muscle weakness in arms and legs.* The patient had two similar, though less severe, episodes over the last 6 months. During this period, she reports to have been anxious and oversensitive to heat. She lost 4 kg of her weight despite maintaining her appetite. She has been constantly feeling hot and sweating. Last night she began to notice a slight weakening of the muscles. On waking up, she could get up only with severe difficulty and was unable to walk. She had no vomiting or diarrhoea. The patient has used Pravastatin (40 mg) because of a family history of dyslipidaemia. She was treated for a toxoplasmosis-chorioretinitis 3 years ago. Family history: her brother had a diagnosis of ankylosing spondylitis. There are no diseases associated with muscle weakness in the family.	
Physical examination:	
BP: 140/70 mmHg; pulse: 100 / min; respiratory rate: 20/min; temperature: 36.6 °C. The skin is warm and moist. Slight hand trembling. Severe proximal muscle weakness; symmetric shortened tendon reflexes. The rest of the physical examination showed no abnormalities.	
Lab tests:	
Hb: 16.7; Ht: 49%; white cell count: 9000; ESR: 1; urea: 13; creatinine: 0.7; sodium: 143; potassium: 2.0; chloride: 108.	

#### Treatment variable: interruptions

**Fig. 1:**
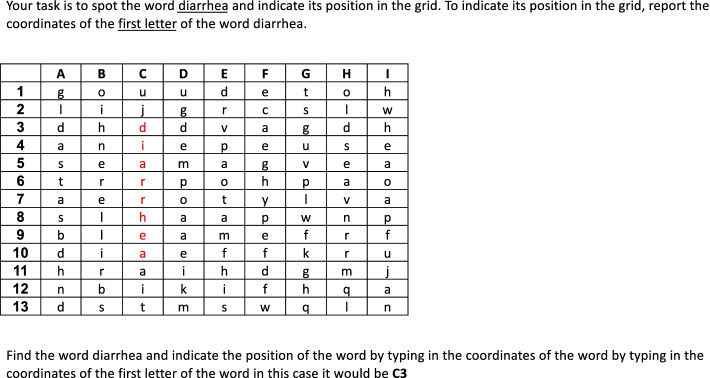
An example of word-spotting puzzle

**Fig. 2:**
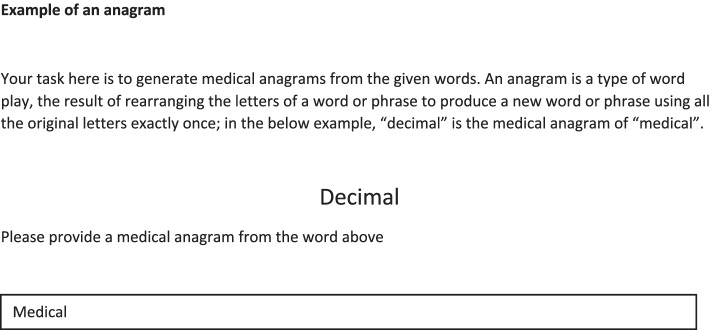
An example of an anagram

There were two types of interruptions utilized in this study. The first was in the form of a word-spotting puzzle (Fig. 1), where the participant were requested to spot a word embedded in a matrix of letters, where the word can appear in horizontal, vertical or diagonal order. The participants were asked to enter the row coordinates corresponding to where the word is spotted. The second form of distraction was generating a medical anagram (Fig. 2) by rearranging the letters of a word provided. Examples of how to solve the puzzle and anagram were provided as part of a training phase.

#### Procedure

The cases were presented, and data were collected using E-Prime 2.0 (Psychology Software Tools, Inc. Pittsburgh, Pennsylvania). Participants were first asked to fill in their basic demographic information to include: sex, age, subspecialty, years from graduation, number of years of experience, and number of hours slept within the last 24 h. This was followed by a welcoming screen stating the aims of the study which was a better understanding of the nature of clinical problem-solving in internal medicine and the effect of time constraints faced by physicians on a daily basis. A screen displaying instructions, with examples, for the task followed. Participants were randomly assigned to receive instructions to diagnose eight cases in one of two counterbalanced orders on a computer. The control group was allowed to diagnose the eight cases without interruptions. Whereas the experimental group was interrupted every other case. The interruption was introduced after displaying the case and allowing 15 s of reading. After solving a puzzle or anagram, participants were returned to the original vignette. They were requested to read the case again and subsequently type the most likely diagnosis. To control for order effects, 4 different versions of the program were prepared so that the sequence of presentation of the cases was reversed. The same was done with the sequence of the condition under which the cases would be solved (i.e., half the participants started diagnosing cases without interruption and the other half started with interruption). Participants were randomly assigned to work with one of the versions of the program. There were no time restrictions for decision making. The computer software automatically recorded time spent to diagnose each case, as well as the participants’ diagnoses.

#### Scoring and analysis

Two experts in internal medicine independently evaluated the diagnoses made by the participants, without being aware of the conditions under which the case had been solved. A diagnosis was considered correct whenever the core diagnosis (e.g., “acute hepatitis” in the case of acute viral hepatitis) was mentioned. If the participant did not cite the core diagnosis, but mentioned a component of the diagnosis (e.g., “myopathy” as the diagnosis in the case of “Hyperthyroidism”), the diagnosis was evaluated as partially correct. Diagnoses that did not fall into one of the previous categories were considered incorrect. Diagnostic accuracy was rated as 2, 1, and 0, respectively for completely correct, partially correct, and incorrect diagnoses. Raters agreed in 84% of their judgments, and differences were solved by discussion. Diagnostic accuracy scores were computed as the mean score per case. Time on task was computed as mean number of seconds per case. Both variables were analyzed using one-way analysis of variance (ANOVA) with interruptions/no-interruptions as the treatment variable (IBM SPSS Statistics 27, Armonk, NY).

### Results

Table 2 contains mean diagnostic accuracy and time-on-task scores for the two conditions of the experiment. No statistical differences between the two conditions of the experiment were found; neither for diagnostic accuracy, *F*(1, 40) = 0.102, *p* = 0.75, nor for time on task, *F*(1, 40) = 0.605, *p* = 0.44. Post-hoc analysis showed that interruption among residents with fewer practice years had no significant effect on diagnostic accuracy.

**Table 2:** Mean and standard deviations of diagnostic accuracy and time-on-task for control (no interruptions) and treatment (interruptions) group in Experiment 1

		N	Mean	Standard Deviation
Mean Diagnostic Accuracy	Control	22	0.91	0.32
	Treatment	20	0.88	0.37
	Total	42	0.89	0.34
Mean Time-on-Task (in seconds per case)	Control	22	319.26	307.86
	Treatment	20	387.73	257.33
	Total	42	351.87	283.60

### Discussion

In this experiment we either interrupted, or did not interrupt residents, engaged in a diagnostic task. We measured diagnostic accuracy and time on task. We were not able to demonstrate effects of interruptions on diagnostic accuracy, nor could an effect be found on the amount of time the residents needed to complete the diagnostic task. Monteiro et al. [26] found a similar lack of evidence regarding effects of interruptions on diagnostic accuracy (although they found a disruptive effect on time on task). Are these findings definitive? Do interruptions not or not convincingly hurt diagnostic decision making? In view of the overwhelming evidence from observational studies that interruptions are endemic in professional practice [20–22] and that they negatively affect surgical [22, 23] and therapeutic [24, 25] procedures, it is hard to believe that they would not influence diagnostic reasoning. Therefore, we had to look at our procedures to find weaknesses that may explain our lack of findings. First, maybe the nature of our interruptions—puzzles and anagrams—were so divorced form medical practice that they failed to have an impact. In addition, we only interrupted half of the cases, which may not have been sufficient for effects to emerge. Second, the interruptions may not have been situated where they hurt most. The reader may recall that the participants were given 15 s to read the case, were interrupted, but subsequently were confronted with the full case again. The latter may have enabled them to recover from the interruption sufficiently to overcome its effect. Finally, despite of the fact that the sample sizes were based on similar studies [10, 11], the study may have lacked sufficient statistical power to detect differences between the conditions of the experiment. Therefore, we conducted a second experiment, repairing these potential shortcomings.

## Experiment 2

In Experiment 2, we increased the number of interruptions, the number of cases, and the number of participants to increase statistical power. More importantly, the interruptions were more ‘lifelike,’ directly relevant to medical practice, and the participants were interrupted in the middle of a case without the possibility to review its first part.

### Method

#### Participants

One-hundred residents were invited to participate. They were enrolled in a 4-year internal medicine residency program at an academic hospital in Saudi Arabia. Eventually, 78 signed up for the experiment. Participants were 22 females and 54 males. Median age was 27 years (SD = 2.20). Mean number of years of clinical practice was 2.20 (SD = 1.41). No significant differences in gender, age and clinical practice were observed between treatment and control groups. Participation was voluntary, without financial incentives, and all participants provided informed consent and anonymity and confidentiality was maintained. Ethical approval to conduct the study was granted by the institutional review board of the King Abdullah International Medical Research Center (KAIMRC), Riyadh, Saudi Arabia.

#### Materials

Participants diagnosed eleven written clinical vignettes that were based on real patient data previously used in published studies [10, 31] and have been shown to be of an intermediate difficulty. Each of the case vignettes consisted of a description of the patient’s medical history and present complaints, physical examination findings, and lab test results. The cases were: Stomach cancer, hyperthyroidism, alcoholic cirrhosis, inflammatory bowel disease, Addison disease, acute viral pericarditis, aortic dissection, acute viral hepatitis, acute myeloid leukemia, pseudomembranous colitis, and acute appendicitis.

#### Interruptions

The interruptions used were common in professional practice. They were unrelated to the case under diagnosis. Each of them required the participant to write a response to a appeal for advice from a colleague, a question of a nurse, a request from a pharmacist, a call for help by an intern regarding a complex differential diagnosis, etc. A full list of the interruptions can be requested from the corresponding author. An example is this:

The intern brings you new information on a 65-year-old male who came to the emergency department with a chief complaint of hematuria. He has a history of diabetes mellitus and hypertension from 10 years ago and diarrhea from 3 days ago. The serum creatinine is 2.5 mg/dL. Your working diagnosis was acute renal failure with intrinsic causes. The student says the patient has now revealed a history of vomiting. He is in doubt whether this finding doesn’t make the diagnostic hypothesis less probable.

What would you tell the intern about the likelihood of the diagnosis?

#### Procedure

The study was conducted in a computer laboratory. Participants were randomly assigned to one of two conditions (control vs interrupted) and each condition group was subdivided into two subgroups; the only difference was the sequence of the cases displayed where one group had the cases displayed in the reverse sequence of the other. The interrupted group comprised 39 participants, and the control group comprised 37 participants. Cases were presented to the participants online using QUALTRICS software (Qualtrics, LLC, Provo, UT, USA), a web-based testing system. The computer laboratory’s computers were pre-set with a link referring to one of the four conditions. Instructions to the participants were provided through the software and they could practice with two example cases before the experiment started. The participants were instructed to provide their responses as quickly as possible, but no measures were taken to enforce this. Each of the case vignettes was presented in two parts on separate pages. The first page contained a description of the patient’s medical history and present complaints, the second page contained physical examination findings, and test results. Interruptions were introduced on a separate page after the patient’s presenting illness and history page and prior to the physical examination findings and test results page. Participants had to write their response to the interruption on the same page. The software did not allow participants to go back to previous pages. After each case space for diagnosis was provided on a separate page. After completing the experiment, demographic data were collected including age, gender, residency level and frequency of encountering similar cases.

#### Scoring and analysis

Scoring and analysis were similar to Experiment 1.

### Results

Table 3 contains mean diagnostic accuracy and time-on-task scores for the two conditions of the experiment. No statistical differences between the two conditions of the experiment with regard to diagnostic accuracy, *F* (1, 76) = 0.016, *p* = 0.90. The interrupted group however needed significantly more time to complete the diagnostic task, *F* (1, 76) = 22.74, *p* < 0.001. Post-hoc analysis showed that interruption among residents with fewer practice years had no significant effect on diagnostic accuracy. There was however an effect on time on task for this group (it needed more time).

**Table 3:** Mean and standard deviations of diagnostic accuracy and time-on-task for control (no interruptions) and treatment (interruptions) group in Experiment 2

		N	Mean	Standard Deviation
Mean Diagnostic Accuracy	Control	37	0.94	0.38
	Treatment	41	0.95	0.32
	Total	78	0.95	0.35
Mean Time-on-Task (in seconds per case)	Control	37	505.34	198.36
	Treatment	41	698.69	159.19
	Total	78	606.97	202.49

### Discussion

Despite increasing the number of interruptions, the number of cases, and the number of participants, and making the interruptions more directly relevant to medical practice, the results of this experiment looked quite similar to those of Experiment 1, with one exception: Participants who were interrupted needed more time to diagnose the cases. These findings are in line with those of Monteiro et al. [26], who also found an effect on time-on-task but failed to find an effect on diagnostic accuracy. In all these studies—the ones reported here and the Monteiro and colleagues' [26] study—participants were encouraged to work fast, but no additional measures were taken to ensure that they experienced time pressure. However, one could argue that in the reality of clinical practice physicians are under constant time pressure while having to make decisions. In fact, experienced time pressure is seen by many health professionals as a major disruptive element of professional practice [32, 33]. Therefore, in Experiment 3, we increased time pressure on our participants.

## Experiment 3

In another line of research on factors that negatively affect diagnostic reasoning, our research group successfully implemented a procedure putting time pressure on participants [10, 11]. This procedure led participating residents into believing that during the experiment they came under ever increasing pressure to complete the tasks. The expectation was that increased time pressure would hurt diagnostic performance of the group that was interrupted most.

### Method

#### Participants

We approached 62 level three senior emergency medicine residents enrolled in the program of the Saudi Board of Emergency Medicine. However, due to various clinical and academic duties scheduling problems of the residents, we were able to recruit only 30 residents from eight hospitals in Riyadh, Saudi Arabia. They were enrolled in a 4-year emergency medicine residency program at an academic hospital in Saudi Arabia. Participants were 8 females and 24 males. Median age was 28 years (SD = 3.44). Mean number of years of clinical practice was 2.38 (SD = 0.91). No significant differences in gender, age and clinical practice were observed between treatment and control group.

#### Materials

The cases used were identical to those used in Experiment 2.

#### Interruptions

The interruptions provided were the same as in Experiment 2.

#### Procedure

The procedure was the same as in Experiment 2. However, in this experiment we explicitly introduced time pressure (in both conditions) to see whether the combination of interruptions and time pressure (only in the treatment group) would negatively affect diagnostic accuracy and time-on-task. Both groups received the following instructions informing them about their work progress:

After each case you have diagnosed, you will receive information about how much work still needs to be done and how much time is left for doing so. If time is running short, you can adapt by working your way faster through the next cases. The number of cases still to be seen is represented by a GREEN bar, whereas the time left is shown as a RED bar. By comparing the two bars, you can see how much time you still have, but the computer will in addition provide you with automated feedback about your progress.

**Fig. 3:**
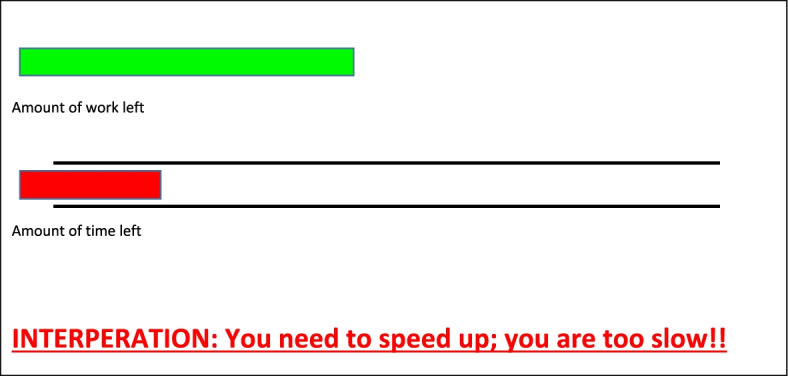
An example of the on-screen visual cues and textual feedback used in Experiment 3 encouraging participants to work as fast as possible. Seen by both groups

Both groups were exposed to the effect of time-pressure through displaying two bars on the screen after each case (Fig. 3). A green bar showed how many cases remained to be seen, and a red bar showed how much time remained. It is important to note that the information displayed by the bars was independent of the actual performance of the resident. The bars were intended to put the residents under time pressure through showing them that they were lagging behind schedule. In addition, textual feedback was provided with the two bars to notify the residents that they were falling behind. The following are examples of the textual feedback provided:

- You are quite fast, but it is not sufficient.
- You are fast, but you still spent more time than was allocated for the first two cases.
- Fast, but you are still behind schedule.
- You are not fast enough; there are still three cases to be seen!

#### Scoring and analysis

Scoring and analysis were similar to Experiment 1 and 2 with one exception. It was decided to award 1 point for each diagnosis that was either entirely or partially correct, and 0 points for incorrect diagnoses.

### Results

Table 4 contains mean diagnostic accuracy and time-on-task scores for the two conditions of the experiment. No statistical differences between the two conditions of the experiment with regard to diagnostic accuracy, *F* [1, 30] = 1.58, *p* = 0.22. The interrupted group needed more time to complete the diagnostic task, but this effect did not reach significance, *F* [1, 30] = 2.37, *p* = 0.14.

**Table 4:** Mean and standard deviations of diagnostic accuracy and time-on-task for control (no interruptions) and treatment (interruptions) group in Experiment 3

		N	Mean	Standard Deviation
Mean Diagnostic Accuracy	Control	15	0.37	0.08
	Treatment	15	0.42	0.12
	Total	30	0.40	0.11
Mean Time-on-Task (in seconds per case)	Control	15	82.22	25.02
	Treatment	15	97.55	29.39
	Total	30	89.89	27.93

### General Discussion

Three experiments were conducted to assess the extent to which interruptions negatively affect diagnostic decision making and time-on-task among physicians. There is a literature showing that health professionals are interrupted often while engaged in providing health care [12, 15, 16]. In addition, interruptions have been demonstrated to affect surgical [22, 23] and therapeutic [24, 25] procedures. It is therefore not unlikely that they may influence diagnostic reasoning as well, although studies demonstrating a causal effect of interruptions on diagnostic accuracy (a token for the quality of diagnostic reasoning) are largely lacking. In the domain of medicine, only one study stands out. Monteiro et al. [26] failed to find an effect on diagnostic accuracy (although time on task was demonstrated to be significantly affected). On the other hand, laboratory studies in psychology provide extensive support for the idea that due to limited capacity of cognitive resources, interruptions may lead to error [18, 29, 34].

Our experiments appear to support the idea of Croskerry’s “cognitive firewalls” that participants may have created to avoid cognitive errors [9]. Participants may have also formed a preliminary diagnosis from the history and present complaints depending on internal schemas that was only supported by data later presented thereby minimally affected by the interruption as the clinical & test findings. However, dependence on internal schemas is more typical of experienced physicians but no significance difference was noted between junior and senior residents.

In the experiments reported here, we have studied the issue using a panoply of methods. In the first experiment, residents were interrupted by a second task consisting of word-spotting puzzles and medical anagrams. Given the failure to find an effect, we concluded that this task was perhaps insufficiently immersive. In the second experiment, we therefore, introduced interruptions that were more lifelike, directly relevant to medical practice. In addition, the participants were interrupted in the middle of a case without the possibility to review its first part. Finally, we increased the number of cases, interruptions, and participants. Again, we failed to find an effect on diagnostic accuracy, although participants took longer diagnosing the cases. In the Discussion section of Experiment 2, we argued that perhaps time pressure is a decisive factor. Without it, physicians who are interrupted may still have sufficient cognitive resources available to recover from it. In Experiment 3, we introduced a procedure that had been demonstrated to be effective in inducing time pressure in residents [10, 11]. We time-pressured both the interruptions and the no-interruptions group. Again, we failed to find the expected effects. Reviewing our overall findings, a number of observations can be made. The first is that they tend to concur with those of Monteiro et al. [26]. This applies in particular to diagnostic accuracy, but time-on-task was in all three experiments higher for the interrupted group (although only significantly so in Experiment 2). The second is that irrespective of the academic background of the residents (internal medicine, emergency medicine) or their level of expertise, expressed in years of practice, their level of performance on the diagnostic task was quite similar (correcting for differences in scoring). On the other hand, time on task varied immensely, from one and a half minute per case to more than 10 minutes. It is unclear why these differences emerged. The test leaders of Experiment 2 (M.A. and N.S.) noticed that they encountered their participants in the middle of examinations. Maybe they were in ‘exam mode” and took the experimental task extremely seriously, thereby overriding any effect of the experimental treatment. It is also plausible that the artificial computer lab environment, though useful in minimizing confounding factors and allowing setting standardization for more robust experimentation, may have oversimplified the complex environment of clinical practice. Such experimental settings strip the environment from contextual factors, such as emotional reactions and behavioral inferences, which have been speculated to affect clinical reasoning processes and potentially, in turn, diagnostic accuracy [35, 36]. Interruptions read on a computer screen, even if detailed, fail to mimic the emotional stress, often found in clinical settings with high interruption incidence, that can contribute to the cognitive burden of physicians that may have contributed to failure to detect an effect in our experiments.

## Conclusion

It seems that within the experimental paradigm reported here, and the Monteiro et al. [26] study, effects of interruptions on diagnostic reasoning are not to be expected anymore. Investigators in this field should look for other approaches to the problem if they still believe that interruptions in the realm of medicine are an important source of error. Second, our findings suggest that accidental environmental events may interfere with experiments in this domain to an extent not previously observed.

## Data Availability

Study datasets are available from the corresponding author upon reasonable request.
